# Decreased telomerase activity and shortened telomere length in infants whose mothers have gestational diabetes mellitus and increased severity of telomere shortening in male infants

**DOI:** 10.3389/fendo.2024.1490336

**Published:** 2024-12-16

**Authors:** Shuhua Liu, Liping Xu, Yan Cheng, Dehong Liu, Bin Zhang, Xianxia Chen, Mingming Zheng

**Affiliations:** ^1^ Department of Obstetrics and Gynecology, Hefei Maternal and Child Health Hospital, Hefei, China; ^2^ Department of Obstetrics and Gynecology, Anhui Women and Children’s Medical Center, Hefei, China; ^3^ Department of Obstetrics and Gynecology, Maternal and Child Medical Center of Anhui Medical University, Hefei, China; ^4^ Fifth School of Clinical Medicine, Anhui Medical University, Hefei, China

**Keywords:** TL, telomerase, TE, telomere length, gestational diabetes mellitus, GDM

## Abstract

**Objective:**

Gestational diabetes mellitus (GDM) is a common complication during pregnancy and increases the risk of metabolic diseases in offspring. We hypothesize that the poor intrauterine environment in pregnant women with GDM may lead to chromosomal DNA damage and telomere damage in umbilical cord blood cells, providing evidence of an association between intrauterine programming and increased long-term metabolic disease risk in offspring.

**Methods:**

We measured telomere length (TL), serum telomerase (TE) activity, and oxidative stress markers in umbilical cord blood mononuclear cells (CBMCs) from pregnant women with GDM (N=200) and healthy controls (Ctrls) (N=200) and analysed the associations of TL with demographic characteristics, biochemical indicators, and blood glucose levels.

**Results:**

The length of telomeres in umbilical CBMCs in the GDM group was significantly shorter than that in the Ctrl group (P<0.001), and the shortening of telomeres in male infants in the GDM group was more significant than that in the Ctrl group (P<0.001) after adjustment for Pre-pregnancy body mass index (PBMI), Pregnancy weight gain (PGW), and Triglyceride (TG) as confounding factors. In addition, the TE expression level in the GDM group was lower after adjustment. There was no statistically significant difference in oxidative stress hydroxydeoxyguanosine (8-OHdG), malondialdehyde (MDA) and superoxide dismutase (SOD) between the two groups. TL was positively correlated with TE activity, and both were negatively correlated with blood glucose levels. There was no correlation between TL and Gestational age (GA), PBMI, PGW, or TG levels.

**Conclusion:**

The poor intrauterine environment in pregnant women with GDM increases telomere attrition and reduces TE activity, which may be potential genetic risk factors for an increased risk of metabolic diseases in offspring later in life due to intrauterine reprogramming.

## Background

In recent years, the global trend towards GDM has significantly increased, especially since the end of the last century (1). According to the International Diabetes Federation (IDF), the global incidence of GDM was approximately 14% in 2021, with standardised prevalences of 12.7%, 9.2%, and 14.2% in low-, middle-, and high-income countries, respectively, with different economic levels (2). Notably, the incidence of GDM in China is approximately 11.91%, which is significantly higher than that in neighbouring Asian countries such as Korea, Japan, and Thailand, where the prevalence of GDM remains below 8% (3).

With advancements in modern medical knowledge, people are becoming increasingly aware of the importance of the unfavourable intrauterine environment in pregnant women with GDM for foetal growth and development. GDM has been shown to be strongly associated with long-term metabolic imbalances in offspring (4–19), including impaired glucose tolerance, obesity, and cardiovascular disease, and these adverse outcomes are proportional to the duration of intrauterine exposure for the foetus (20–24). A variety of factors contribute to the poor intrauterine environment, prompting the foetus to adapt to environmental changes through a “foetal programming” mechanism, a process that may increase the risk of future diseases in the short or long term. Evidence suggests that children of women with diabetes, particularly those with type 1 diabetes, may be at increased risk of glucose intolerance and cardiovascular disease, even in infancy and adolescence (20). Notably, GDM can contribute to an undesirable intrauterine environment; even if the mother successfully controls her blood glucose levels during pregnancy, she may still experience adverse pregnancy outcomes and long-term health problems in her offspring (25). These phenomena may be related to changes in genetic pathways and endocrine regulation, leading to adaptive reprogramming in foetuses exposed to adverse intrauterine environments, which may be a critical pathway for the development of metabolic diseases in adulthood (26, 27). Although these studies have shed light on some of the causes of disease associations, the specific mechanisms underlying the increased risk of chronic diseases caused by intrauterine exposure to adverse environments are still largely being explored and have not been fully elucidated (28). Changes in the telomere/telomerase system in this complex adaptive programming process have long been relatively underappreciated. Telomeres, which are composed of noncoding DNA repeats at the ends of chromosomes (the basic unit of which is TTAGGG), have variable lengths and are responsible for protecting chromosomes and maintaining genome integrity (29, 30). Typically, the length of telomeres in somatic cells decreases with age (31). During cell division and chromosome replication, approximately 150 to 200 base pairs are reduced at a time, and if this process continues, DNA damage accumulates; once telomeres shrink to a critical length, cells begin the ageing process (32). Thus, telomeres are often seen as markers of cumulative cellular damage (29, 30). To date, an abnormal TL has been found to be associated with a wide range of diseases, including impaired glucose tolerance, dyslipidaemia, arteriosclerosis, obesity, diabetes, cardiovascular disease, and many cancers (33).

TL adjustment is a complex process influenced by multiple factors that involves internal and external elements. Among these factors, TE activity plays a key role in the regulation of TL (34). Studies have shown that the rate of telomere shortening in populations of African descent exceeds that in populations of European descent (34). Additionally, single-nucleotide polymorphisms in the TE reverse transcriptase (hTERT) promoter region unique to the Japanese population lead to effective resistance to the increasing phenomenon of telomere loss with age (35), an effect not reported in other ethnic groups (36). TE is responsible for telomere elongation and is a basic ribonucleic acid-dependent DNA polymerase. TE uses its own telomerase RNA component (TERC) to cooperate with telomerase-associated proteins (human) via its RNA template under the catalysis of the TE catalytic subunit human telomerase reverse transcriptase (hTERT). Together with telomerase-associated protein 1 (TEP1), a complex reverse transcriptase complex that uses an RNA template to reverse transcribe DNA repeats catalysed by TERT to produce DNA repeats is formed, which are added to telomere ends to compensate for shortening due to the deletion of replication ends (37, 38). Previous sporadic studies on the effects of gestational diabetes on the telomere system in neonates have been inconclusive. Considering the adverse intrauterine environment caused by GDM, we speculate that GDM may have a certain effect on TL and TE activity in newborns, and we conducted our research on the basis of this hypothesis.

## Materials and methods

### Study participants

This study adopted a prospective cohort study design and focused on a group of pregnant women whose files were established and who received regular prenatal check-ups at Hefei Maternal and Child Health Hospital from November 2022 to October 2023. We systematically collected the basic information of these pregnant women (including their demographic and clinical characteristics) and built an electronic health record database. Over the course of the study, we continued to follow up on the pregnancy progress, pregnancy outcomes, and newborn birth status of the participating pregnant women. This research protocol was successfully approved by the Ethics Review Committee of Hefei Maternal and Child Health Hospital. Before each pregnant woman was enrolled in the study cohort, we detailed the purpose and importance of the study and ensured that they signed a written informed consent form on the basis of their full understanding.

The inclusion criteria were as follows: ≥18 years of age, singleton pregnancy, establishment of a file at 8-12 weeks of gestation, prenatal examination and delivery at Hefei Maternal and Child Health Hospital, and oral glucose tolerance test (OGTT) with 75 grams of glucose conducted between 24 and 28 weeks of gestation. The exclusion criteria were as follows: pregnant women with multiple pregnancies; pregnant women who underwent therapeutic labour induction because the foetus was malformed or stillborn; pregnant women with a history of drug use, malignancy, essential hypertension, type 1 or type 2 diabetes, liver insufficiency, renal insufficiency, autoimmune diseases, long-term use of drugs that affect blood sugar, etc.; and pregnant women with incomplete information provided in their individual medical records.

The diagnostic criteria for GDM were based on the criteria of the International Diabetes and Pregnancy Study Group For the OGTT conducted between 24 and 28 weeks of gestation, 75 grams of glucose were taken orally, and fasting blood glucose (FPG), 1-hour blood glucose, and 2-hour blood glucose levels were measured. If any of the following criteria were met, GDM was diagnosed: a FPG level ≥ 5.1 mmol/L, a 1-hour plasma glucose level ≥ 10.0 mmol/L, or a 2-hour plasma glucose level ≥8.5 mmol/L.

### Specimen collection

After delivery of the foetus and placenta, a sample of umbilical cord blood was collected by a specialist obstetrician via an EDTA anticoagulant tube according to standard protocols in the field of obstetrics. The umbilical cord blood sample was subsequently left in its native state for two hours and centrifuged at 3000 rpm for ten minutes to achieve effective separation of plasma from monocytes. The isolated plasma and blood cells were carefully aliquoted into 2 mL EP tubes and stored at a cryogenic temperature of -80 degrees Celsius prior to DNA extraction. Total DNA was isolated from monocytes via the use of ATL, proteinase K, and RNase A buffers (Qiagen, Inc., Valencia, CA) for phenol–chloroform extraction and ethanol precipitation.

#### Determination of TL

TL is determined via a method based on fluorescence quantitative PCR proposed by Cawthon, which is low-cost and relatively simple method that is commonly used for determining TL. In this method, the relative TL of different samples was obtained by measuring the T/S ratio of the telomere copy number (T) and single-copy gene (S) of the sample and reference DNA, which is proportional to the average TL. In this study, the reference gene was selected as a single copy of 36B4, the sequence of which is reported in the reference article (39, 40), and the primers used for telomere detection are reported in the reference report (40). Amplification was performed on an ABI 7900 real-time PCR instrument (Applied Biosystems, Foster City, California) via an optimised program, and the melting curve program was used. The telomeric gene primers were as follows: tel F1: CGGTTTGTTTGGGTGGGTTTGGGTTTGGGTTTGGGTTTGGGTT; tel R1: GGCTTGCCTTACCCTTACCCTTACCCTCTTACCCTTCT. The primers for the internal control genes were as follows: 36B4-F: CAGCAAGTGGGAAGGTGTAATCC; and 36B4-R: CCCATTCTATCATCAACGGGTACAAA. The quantitative PCR procedure was as follows: 95°C for 1 min; 40 cycles at 95°C for 5 s and 55°C for 45 s. The data quality control measures were as follows: The final Ct value of each detected gene was taken as the average of the Ct values of the two replicate wells. For the internal control gene, if the coefficient of variation of the Ct value of the two replicates was greater than 2.5%, the data of this locus were excluded. For telomere genes, if the coefficient of variation of the Ct value of two double wells was greater than 5%, the data of this locus were excluded. If there was a large difference in the Ct values between the two replicates, the data for that locus were invalidated. For the final Ct value of each detected gene, the difference between the CT value of the negative parameter and the CT value of the sample should be greater than 4 (that is, the Ct negative parameter - Ct sample≥4); otherwise, the data of this locus were considered invalid. The relative telomere length was calculated as follows: TL (T/S) = 2-ΔΔCt, where ΔCt = Mean Ct (telomere) - Mean Ct (internal reference gene) and ΔΔCt =ΔCt (sample to be tested) -ΔCt (corrected sample).

#### Determination of TE activity and oxidative stress indicators

An ELISA kit (ELISA, China) was used to measure the TE activity and oxidative stress (SOD, 8-OHdG, and MDA) levels. The absorbance (OD value) was determined with a microplate reader at a wavelength of 450 nm, and the amount of protein of interest in the sample was calculated from a standard curve. The data analysis steps were as follows: 1) By default, each detected protein was read in 2 parallels; each parallel well was read 3 times and corrected with blank wells; the average of the 3 readings was calculated as the absorbance value of each parallel well; and the average value and coefficient of variation of the 2 parallels were calculated. If the coefficient of variation exceeded >15%, the data were highly variable and may be biased. 2) A standard curve was drawn according to the concentration and absorbance value of the standard, and the protein concentration of the sample to be tested in the corresponding plate was calculated according to the standard curve of each plate. 3) When the absorbance value of the sample to be measured exceeded the highest point of the standard curve, the sample was diluted to the linear range and remeasured, and the corresponding dilution factor was multiplied when the concentration was calculated.

### Statistical methods

SPSS 26.0 statistical software (IBM, New York, USA) was used for analysis. For a distribution that conforms to normality, it is expressed as a mean ± standard deviation. When the data does not conform to a normal distribution, median and quartile ranges are used to describe trends in the dataset and dispersion. Independent-samples T-test for variables that conform to normal distribution and satisfy the assumption of variance homogeneity. When the non-normal distribution or the homogeneity assumption of variance is not satisfied, the Mann-Whitney U test is used for comparison between groups, and the analysis of covariance (ANCOVA) is used to control for confounders. The results of the analysis were visualized using R software (R-4.3.2, UoA, New Zealand). Spearman was used to analyse the correlation between TL and clinical indicators. The difference is considered statistically significant when the calculated P < 0.05.

## Results

**Figure 1:**
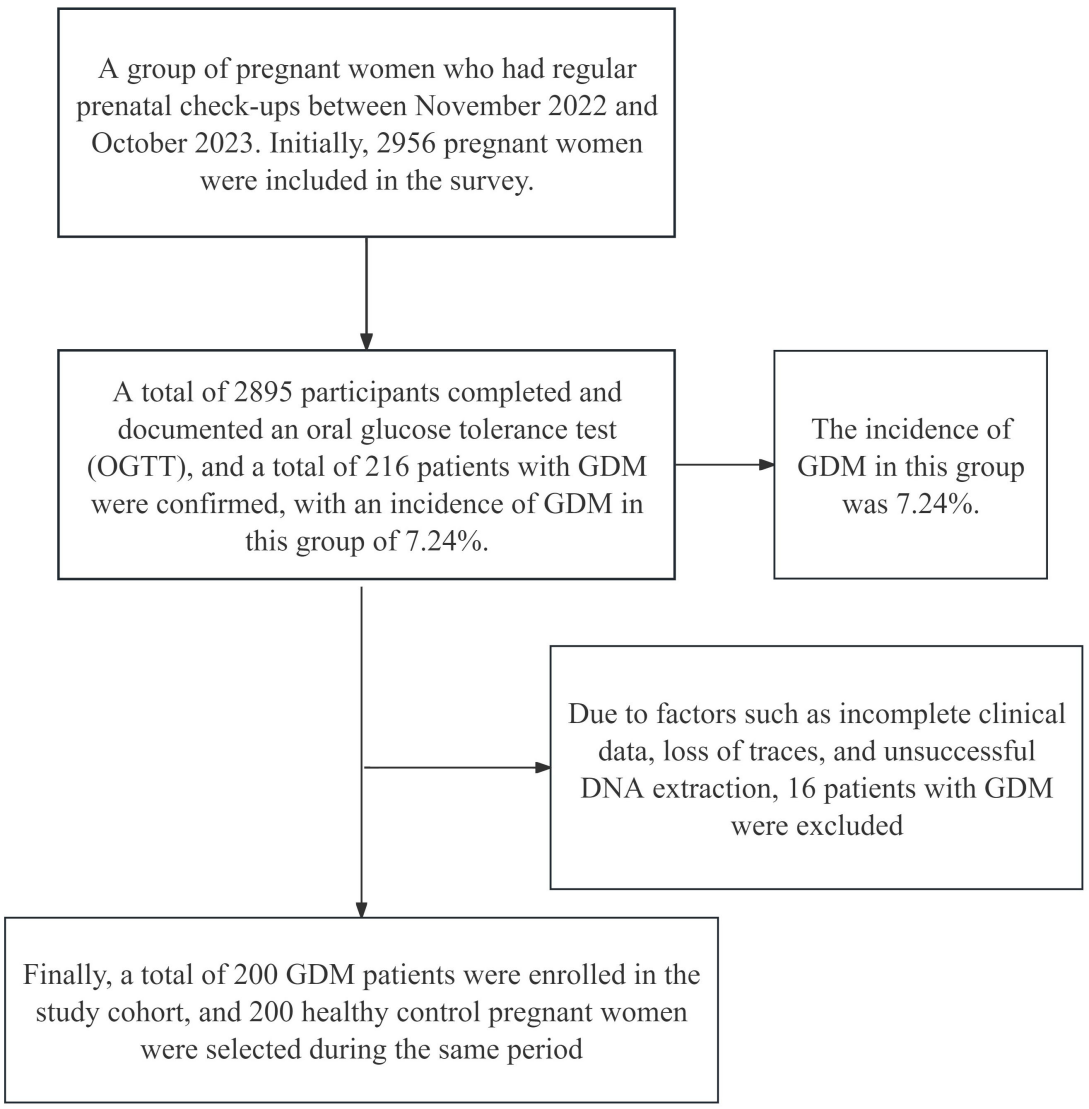
Flowchart of the research object.

The flowchart is shown in **Figure 1**. This study included a group of pregnant women whose files were established at Hefei Maternal and Child Health Hospital and who received regular prenatal check-ups from November 2022 to October 2023. Initially, 2956 pregnant women were surveyed, and these women were included in the study. Among these women, 2895 completed an oral glucose tolerance test (OGTT) and had documented results. A total of 216 patients were diagnosed with GDM, with an incidence of 7.24% in this population. Sixteen patients were subsequently excluded because of factors such as incomplete data, loss of tracking, and unsuccessful DNA extraction. Finally, a total of 200 GDM patients were enrolled in the study cohort, and 200 Ctrls who visited during the same period were selected.

### Comparison of demographic and clinical data between pregnant women with GDM and Ctrls without GDM

**Table 1** shows that the PBMI, PWG and TG levels of the GDM group were significantly greater than those of the Ctrl group (P<0.05). The results of the OGTT revealed that the 1-hour plasma glucose and 2-hour plasma glucose levels in the GDM group were significantly greater than those in the Ctrl group. The gestational age at delivery was earlier in the GDM group than in the Ctrl group.

**Table 1:** Comparison of demographic information and clinical indicators between pregnant women with GDM and Ctrl group.

Indicators	GDM group (N=200)	Ctrl group (N=200)	*P*
Maternal age (years)	30.21±3.76	30.87±3.97	0.089
PBMI (Kg/m^2^)	22.88±3.37	20.86±2.34	**<0.001**
PWG (kg)	15.39±5.13	13.71±5.40	**0.002**
OGTT FPG (mmol/1)	5.22±0.56	4.55±0.3	**<0.001**
OGTT 1h (mmol/1)	9.39±1.83	7.4±1.29	**<0.001**
OGTT 2h (mmol/1)	7.88±1.42	6.33±1.02	**<0.001**
GA (weeks)	39.27±0.87	39.54±0.9	**0.002**
LDL (mmol/L)	3.48±1.09	3.38±0.79	0.355
HDL (mmol/L)	1.85±0.34	1.93±0.36	0.051
TG (mmol/L)	3.2±1.41	2.8±1.29	**0.005**
TC (mmol/L)	5.91±1.39	5.89±1.03	0.891
SBP (mmHg)	118.32±9.94	116.89±9.84	0.149
DBP (mmHg)	77.75±7.41	76.48±6.77	0.075
PPH (ml)	310.05±145.24	304.43±154.66	0.708
NBW (g)	3361.09±376.85	3393.76±430.06	0.422
Postpartum complications (Yes/No)	171/29	172/28	0.494
Newborn gender (Boy/Gril)	110/90	100/100	0.389
Apgar Score (9-10)	200	200	/

GDM, Gestational diabetes mellitus; Ctrl, Healthy control; PBMI, Pre-pregnancy body mass index; PWG, Pregnancy weight gain; GA, Gestational age; OGTT FPG, glucose tolerance test and fasting blood glucose; LDL, Low Density Lipoprotein; HDL, High-density lipoprotein; TG, Triglyceride; TC, Total Cholesterol; SBP, Systolic blood pressure; DBP, Diastolic blood pressure; PPH, Postpartum hemorrhage; NBW, Newborn birth weight.

Bold fonts indicate statistical differences.

### Comparison of the relative TL of umbilical CBMCs in the GDM and Ctrl groups

**Figure 2:**
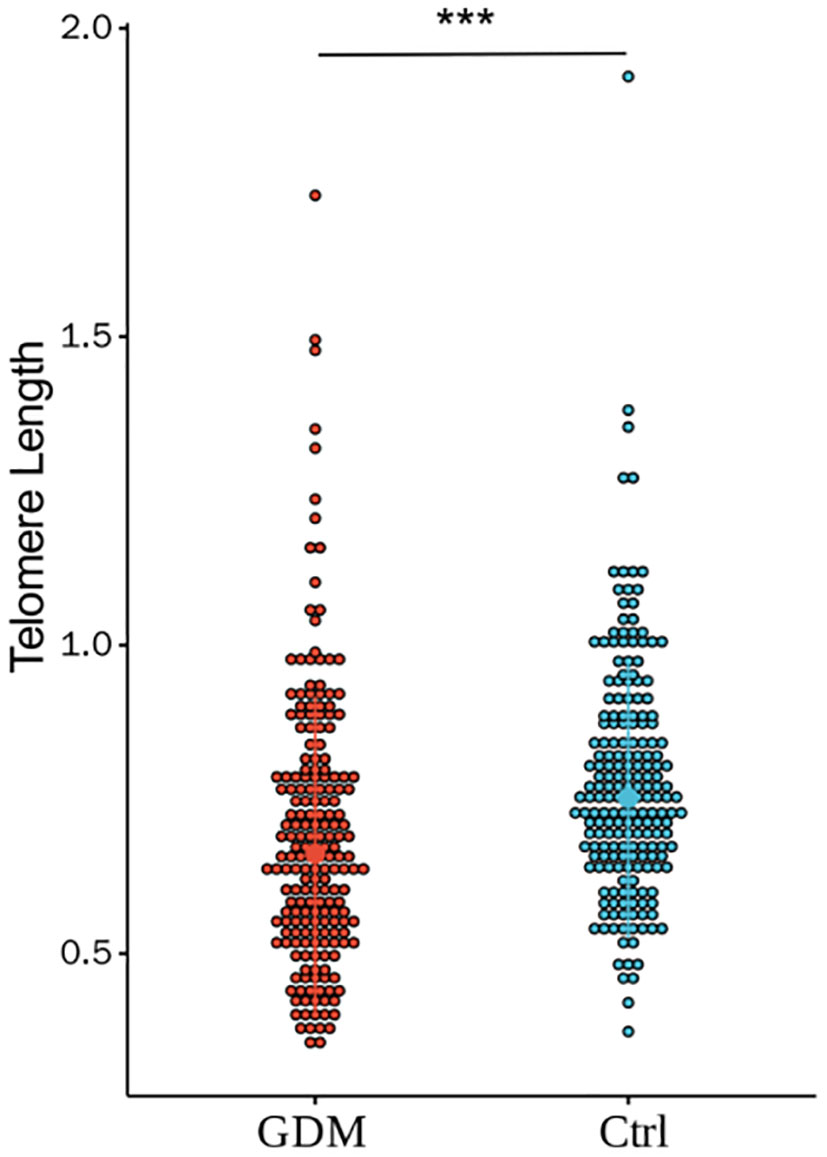
Comparison of the relative TL of umbilical CBMC in GDM group and Ctrl group.(*** for P<0.001) There was a significant statistical difference in relative telomere length between the two groups (0.69±0.22 vs. 0.77±0.19, P < 0.001). By adjusting for PBMI, PWG, and TG, there were still differences between the two groups (P<0.001).(*** for P<0.001, Ctrl for healthy control).

**Figure 3:**
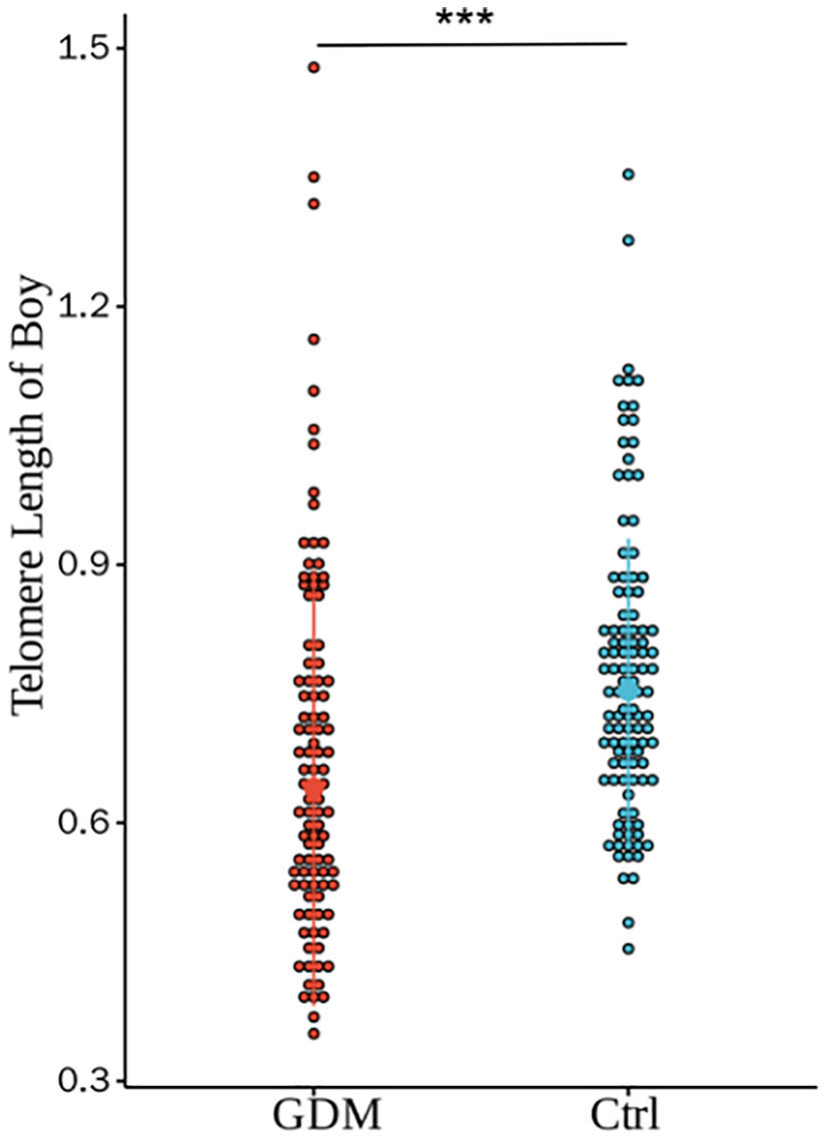
Comparison of umbilical cord blood relative TL between GDM group and Ctrl group offspring male infants. There were statistically significant differences in the relative TL between the two groups (0.68±0.22 vs. 0.78±0.17, P < 0.001), and the differences were still statistically significant after adjusting for PBMI, PWG, and TG (P<0.001).(*** for P<0.001,Ctrl for healthy control).

**Figure 4:**
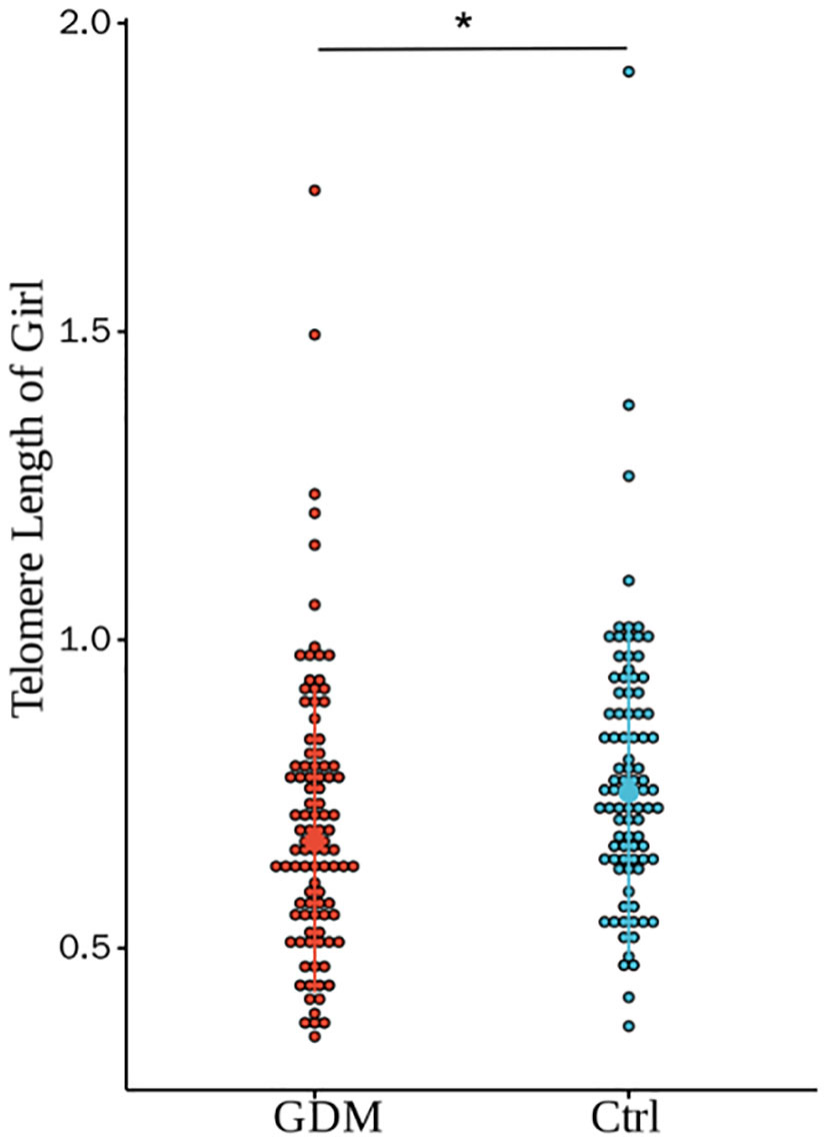
Comparison of umbilical cord blood relative TL of GDM group and Ctrl group offspring of female infants. There was a statistically significant difference in the relative TL between the two groups (0.71±0.23 vs. 0.76±0.22, P=0.028), and the difference was still statistically significant after adjusting for PBMI, PWG, and TG (P=0.322).(* for P<0.05, Ctrl for healthy control).

As shown in **Figure 2**, the relative TL of CBMCs in the GDM group was significantly shorter than that in the Ctrl group, and the difference between the two groups was statistically significant (0.69 ± 0.22 vs. 0.77 ± 0.19, P < 0.001). However, after correction for PBMI, PWG, and TG levels, the difference in TL between the two groups remained statistically significant (P < 0.001). According to the stratification analysis of infants, as shown in **Figures 3**, **4**, there was a statistically significant difference in the relative TL of umbilical cord blood cells between the GDM group and the Ctrl group (0.68 ± 0.22 vs. 0.78 ± 0.17, P < 0.001), and the difference remained statistically significant after adjustment for PBMI, PWG, and TG levels (P < 0.001). There was a statistically significant difference in the TL of umbilical cord blood cells before correction, but no difference was observed after adjustment (P = 0.322).

### Correlation analysis between relative TL and clinical indicators

**Figure 5:**
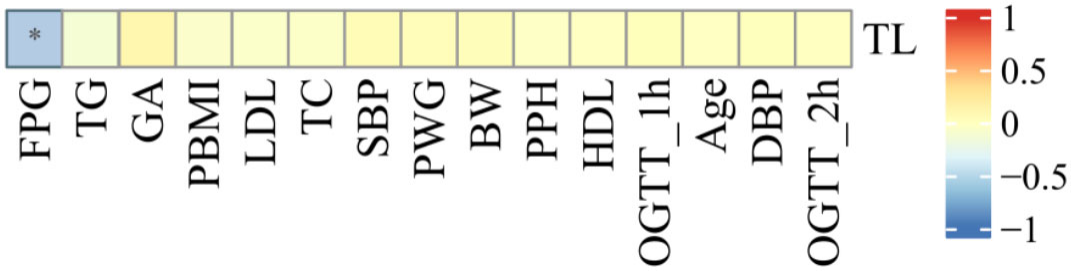
Correlation analysis between cord blood relative TL and clinical indicators. As shown in figure , the relative TL in umbilical cord blood was negatively correlated with FPG (r = -0.140, P = 0.021), and there was no correlation with the statistical differences between PBMI, Maternal age, PWG and TG (all p>0.05). (* for P<0.05).

As shown in **Figure 5**, the relative TL of umbilical CBMCs was significantly negatively correlated with FPG levels determined via an OGTT (r = -0.140, P = 0.021), but it was not correlated with the following indicators: PBMI (r = -0.062, P = 0.311), PWG (r = 0.044, P = 0.469), GA (r = 0.112, P = 0.067), or TG levels (r = -0.049, P = 0.415).

### Comparison of TE expression levels and oxidative stress indices in umbilical cord blood between the GDM group and the Ctrl group

**Figure 6:**
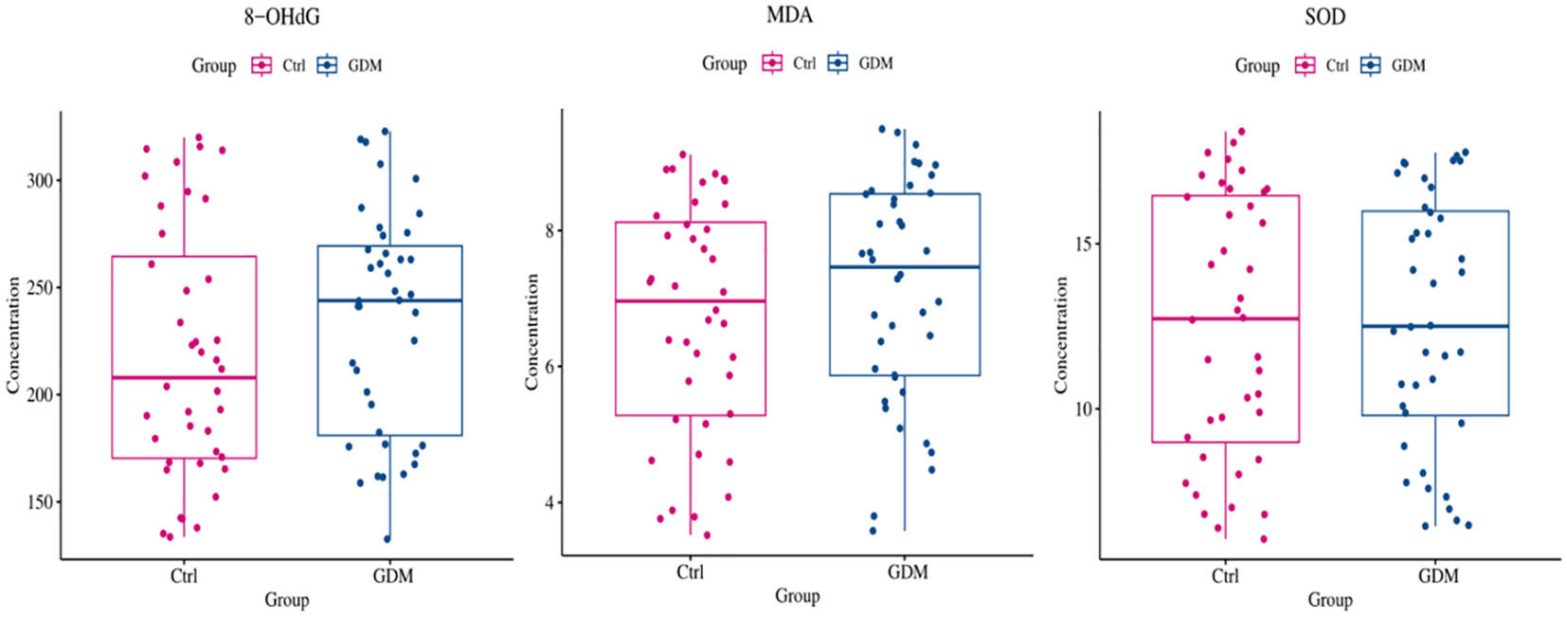
Comparison of oxidative stress indexes in umbilical cord blood between GDM patients and healthy control patients. As shown in figure, although the expression levels of 8-OHdG, MDA, AND SOD in the umbilical cord blood of GDM patients increased, no statistical difference was found (all P>0.05).

**Figure 7:**
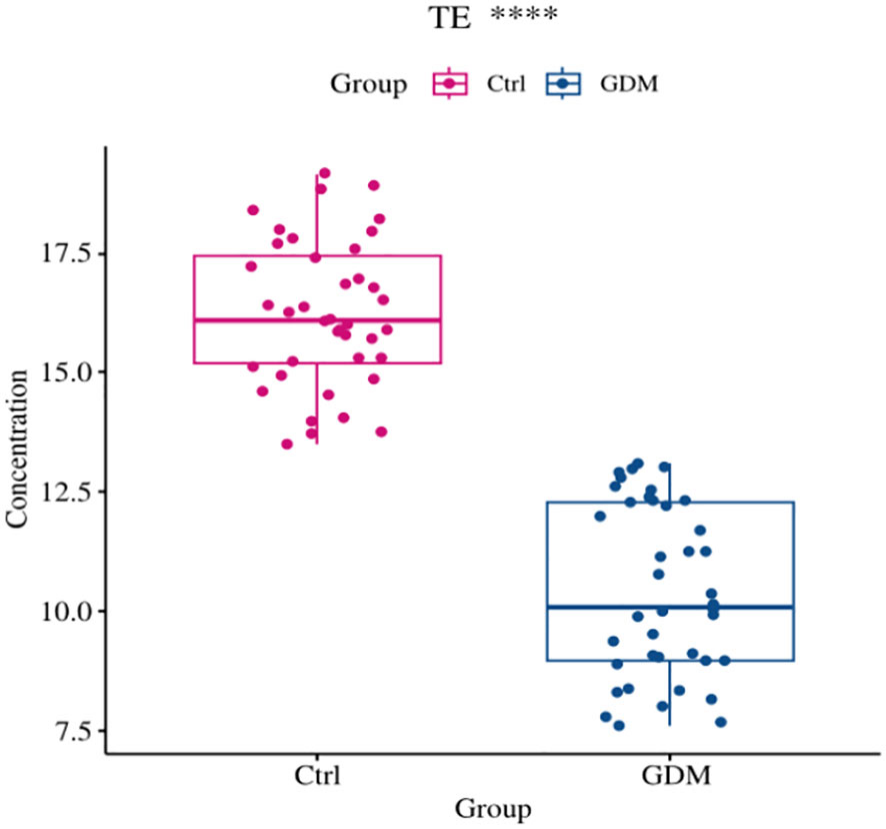
Comparison of cord blood telomerase expression levels between GDM group and Ctrl group. The expression level of TE in cord blood of GDM offspring was significantly lower than that of Ctrl group (10.43±1.80 vs. 16.24±1.53, P<0.001), and the difference was still statistically significant after adjusting for PBMI, PWG, TG (P<0.001).(*** for P<0.001,TE for telomerase, Ctrl for healthy control).

As shown in **Figures 6**, **7**, the expression level of TE in the umbilical cord blood of the GDM group was significantly lower than that in the cord blood of the Ctrl group (P < 0.001), and the difference was still statistically significant after adjustment for different factors (P < 0.001). Although the levels of 8-OHdG, MDA and SOD were significantly increased in the umbilical cord blood of the GDM group, there was no significant difference after adjustment for different factors (all P > 0.05).

### Correlation analysis between TE and TL and various clinical indicators

**Figure 8:**
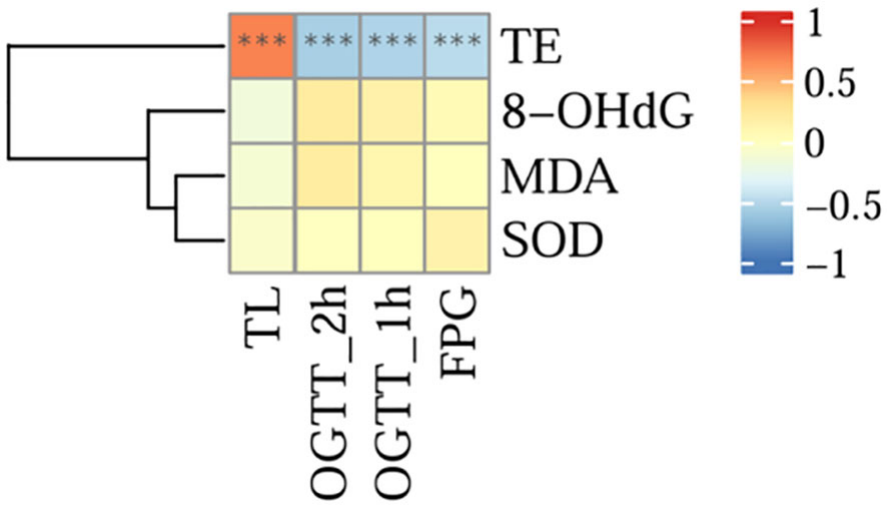
Correlation analysis between TE and TL and various clinical indicators (***for P<0.001).

The results of the analysis are shown in **Figure 8**, and there was a significant positive correlation between TL and the serum TE expression level (r = 0.699, P < 0.001). However, there was a significant negative correlation between TE and OGTT-FPG (r = -0.487, P < 0.001), OGTT-1h (r = -0.538, P < 0.001), and OGTT-2h levels (r = -0.567, P < 0.001).No correlation was found between TL and oxidative stress indicators (8-OHdG, MDA, SOD) (all P>0.05).

## Discussion

In this study, the incidence of GDM in this region was approximately 7.24%, and the relative TL of CBMCs in the offspring of GDM patients was significantly shorter than that in the offspring of healthy pregnant women, and the expression level of TE was reduced. Correlation analysis revealed that there was a positive correlation between TL and TE activity and that there was a negative correlation between the two parameters and blood glucose levels. This finding differs from those of previous studies (41), in which no difference in TL but only a certain change in TE activity were reported. On the one hand, the reason for the difference in results may be that they had a sample size of only a few dozen cases; on the other hand, the reason could be the difference in detection methods, as they used flow cytometry.

Previous studies have suggested that increased foetal oxidative stress in the intrauterine environment is associated with maternal GDM and accelerated telomere attrition (42–44). However, unlike in these studies, in this study, an increase in oxidative stress was not observed in the GDM group, but there was no significant difference compared with that in the Ctrl group, which differs from the findings of previous studies. Therefore, this study suggests that abnormal oxidative stress indicators may be a potential driver of telomere shortening but not a fundamental factor. In addition to cell division, TL may also be regulated by a variety of factors, such as genetic factors and infection, in addition to cell division (45, 46). Telomeric DNA has been shown to be highly sensitive to reactive ROS, especially double-stranded telomeric DNA, which is relatively susceptible to ROS cleavage and has a cleavage preference at the 5’ end of the 5’-GGG-’ sequence (47). Through this study, we speculate that the lack of differences in umbilical cord blood oxidation indices in the offspring of GDM patients may be due to the short duration of GDM and the inherent oxidation and antioxidant balance mechanism in the human body, even in the foetal stage. Thus, the mechanism of telomere shortening may not be solely attributable to the increase in oxidative stress.

Telomeres, as protective structures at the ends of chromosomes, play a key role in maintaining the integrity of DNA information, and their length decreases with age (48–50). In addition, effectively controlling for the confounding factor of TL change may be caused by maternal age (P>0.05). In addition, we also found that the levels of PBMI, PGW, and TG in the GDM group were greater than those in the Ctrl group, and the elevated levels of these indicators were consistent with the typical metabolic abnormalities in GDM patients. Previous studies have shown that PBMI (28, 51–55), PGW (56, 57), and TG (58) levels are factors contributing to telomere shortening in offspring. Therefore, we adjusted for these factors, and the results of the analysis revealed that the TL of umbilical cord blood cells in the offspring in the GDM group was still shorter than that of the offspring in the Ctrl group, and there was a negative correlation with blood glucose levels. In a 2014 study by Xu et al., which showed that the offspring of pregnant women with GDM had relatively short telomeres in cord blood (59), although the factors that may affect TL, such as maternal age and neonatal weight, were adjusted for, factors that may differ in the disease characteristics of GDM patients were not adjusted for, so the results obtained are debatable.

In addition, we analysed the sex stratification of neonates and reported a more pronounced trend towards TL shortening in male neonates, which is consistent with the findings of previous studies (60, 61). Similar studies have also shown that elevated maternal blood glucose levels are associated with an increased risk of impaired glucose tolerance in male offspring (62), and there is evidence that TL in boys is closely related to their glucose load response (63), suggesting that sex differences may affect telomere sensitivity to blood glucose and, in turn, health throughout life (63). However, in 2017, Hjort L et al. evaluated the offspring of pregnant women with GDM from 9 to 16 years of age via quantitative PCR and reported that there was no significant difference in the total number of children with GDM, with only the female offspring showing telomere shortening (64). Because telomere changes are affected by many factors, the telomere state from childhood to adolescence may not directly reflect the intrauterine condition, considering the effects of time and the external environment. Although the results of these studies differ, they all point to the hypothesis that GDM creates an unfavourable intrauterine environment or induces changes in the telomere system, which may be a potential basis for future studies of the pathogenesis of metabolic diseases caused by intrauterine GDM exposure. The association between foetal TL and metabolic phenotype may be regulated by the intrauterine environment, sex-specific physiological mechanisms, and sex-specific genetic and epigenetic alterations, and further research is urgently needed to improve the understanding of this phenomenon (65). In addition, we also observed a decrease in umbilical cord blood TE activity in the offspring of pregnant women with GDM compared with Ctrls, and TL was positively correlated with TE activity. However, both were negatively correlated with glycaemic indicators, which reinforces our focus on the role of the hyperglycaemic environment in regulating the telomere/telomerase system given that gestational diabetes is characterized by hyperglycaemia.

There are shortcomings in this study, and we had no mechanisms for further study of telomere shortening. We did not measure TL in the mothers. In addition, there are a variety of methods for the detection of telomere length and telomerase, and these methods have different advantages and disadvantages. For example, FISH technology still has irreplaceable advantages in some specific application scenarios (such as detailed analysis of telomere length of specific chromosomes in a single cell), but qPCR technology has become the preferred method for detecting telomere length in most research and clinical applications due to its advantages of high throughput, low cost, easy operation, high sensitivity and specificity (66–68). In addition, TRAP technology does have more advantages in the in-depth study of the mechanism of telomerase activity, but the ELISA detection technology of telomerase in serum has also been adopted in many articles (69–71). Therefore, the in-depth study of the mechanism of telomerase activity is the focus of our research group, and we will also use multiple detection methods for verification in the follow-up detection to reduce the bias of the results and achieve more accurate research results.However, our research also has advantages; for one of the important factors affecting TL by maternal age, we reduced the age gap as much as possible to homogenize the samples in the initial study object inclusion stage.

## Conclusions

Overall, this study revealed that the shortening of TL in neonates born to GDM patients was more pronounced in the male infant population, suggesting that intrauterine exposure to GDM in a poor intrauterine environment increased telomere attrition, which may be one of the key mechanisms linking maternal GDM to the increased risk of metabolic disease in offspring later in life, and further longitudinal association studies are needed.

## Data Availability

The datasets presented in this study can be found in online repositories. The names of the repository/repositories and accession number(s) can be found in the article/supplementary material.
